# Recurrent fixation failure of a distal femur fracture associated with intellectual disability: A case report

**DOI:** 10.1016/j.ijscr.2025.111046

**Published:** 2025-02-12

**Authors:** Zaid Sawaftah, Ahmad R. Soboh, Ameer Awashra, Osama Ewidat, Alaa Hmeedan, Qusai Jaludi

**Affiliations:** a Department of Medicine, An-Najah National University, Nablus, Palestine; b Department of Orthopedic and Joint Surgery, Rafidia Surgical Hospital, Nablus, Palestine

**Keywords:** Distal femur fracture, Intellectual disability, Recurrent fixation failure, Advanced fixation techniques, Cognitive impairments

## Abstract

**Introduction and importance:**

Distal femur fractures, particularly in patients with intellectual disabilities, pose unique challenges due to factors such as non-compliance with postoperative instructions and recurrent fixation failures. These complications necessitate multiple surgical interventions, increasing morbidity and disability.

**Case presentation:**

A 59-year-old female with intellectual disability and comorbid diabetes and hypertension sustained a distal femur fracture. Initial management included open reduction and internal fixation (ORIF) with plating. However, due to non-adherence to postoperative instructions, the patient experienced recurrent fixation failures, necessitating two revision surgeries involving long lateral distal femur locking plates and additional stabilization techniques, including cerclage wiring. Despite comprehensive care, the patient faced prolonged recovery and functional limitations.

**Clinical discussion:**

We present the challenges in managing distal femur fractures in patients with cognitive impairments, including non-compliance and biomechanical complications. Also, we highlight the importance of advanced surgical techniques, tailored postoperative care, and close monitoring to prevent recurrent failures. A holistic care approach addressing both physical and cognitive aspects is essential for improving outcomes in this vulnerable population.

**Conclusion:**

Managing distal femur fractures in intellectually disabled patients requires a combination of advanced fixation techniques, enhanced patient and family education, and vigilant follow-up to mitigate complications. This case emphasizes the need for multidisciplinary strategies that integrate psychological resilience into perioperative care to optimize recovery and reduce long-term disability.

## Highlights

• Challenges of managing distal femur fractures in patients with intellectual disabilities
• Recurrent fixation failures necessitated advanced surgical interventions.
• Holistic care approach integrating cognitive and physical aspects improved outcomes.
• Psychological resilience and patient education critical in perioperative care
• The case emphasizes tailored strategies to prevent complications in vulnerable populations.

## 1 Introduction

Distal femur fractures, particularly in patients with unique challenges such as intellectual disabilities and cognitive impairments, present complex treatment scenarios. These fractures are prone to fixation failures due to various factors, including biomechanical stress, bone quality, and difficulties adhering to postoperative protocols. Recurrent fixation failure complicates the treatment further, necessitating repeated surgical interventions and leading to prolonged disability and higher morbidity [1,2].

Herein, we present the case of a 59-year-old female with intellectual disability and a history of diabetes and hypertension who sustained a left distal femur fracture, initially treated with open reduction and internal fixation. However, her cognitive impairment led to non-compliance with postoperative instructions, resulting in recurrent fixation failures and necessitating multiple revision surgeries to restore stability and prevent further complications.

## 2 Case presentation

**Fig. 1:**
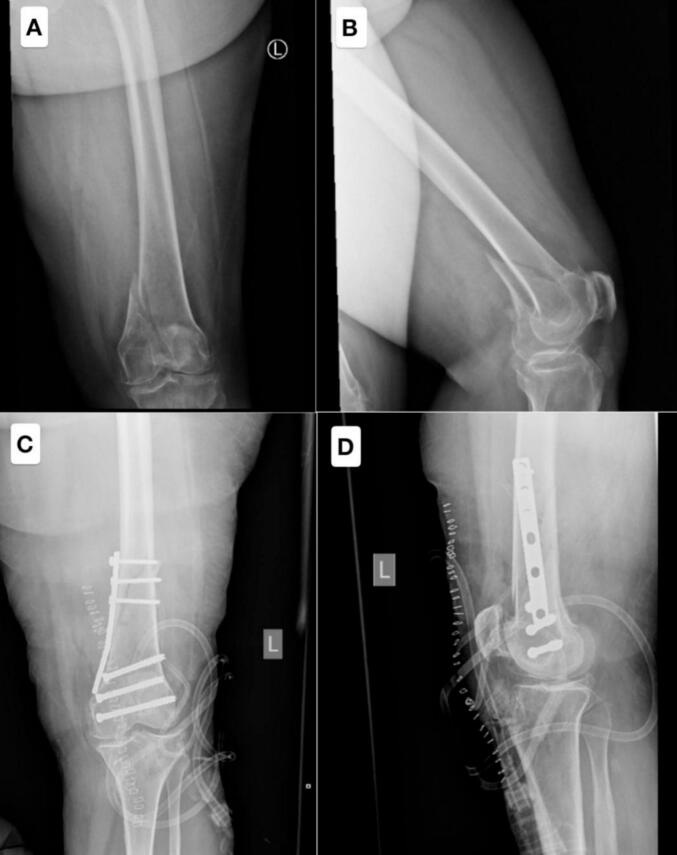
Figs. A and B demonstrate a distal femur fracture with intra-articular extension, highlighting the complexity of the injury. Figs. C and D show postoperative imaging following open reduction and internal fixation (ORIF). The procedure involved the placement of cannulated screws extending from the medial condyle to the lateral condyle, along with an antigliding plate applied to the medial aspect of the distal femur for stabilization. However, the postoperative X-ray revealed that the fracture had extended proximally, indicating a more extensive injury than initially anticipated.

A 59-year-old female patient with a significant medical history of mental retardation, cognitive impairment, diabetes mellitus (DM), and hypertension (HTN), who had not received any form of care or management for her mental health conditions, was admitted with a diagnosis of a left distal femur fracture (shown in Fig. 1).

The patient presented with complaints of left knee pain, localized to the distal femur, which worsened with knee movement and was accompanied by an inability to bear weight. On physical examination, there was tenderness and mild swelling of the distal thigh and knee, but no ecchymosis or evidence of varus or valgus deformity. A detailed neurovascular examination revealed no abnormalities.

The fracture was initially stabilized with a long back slab, and the patient was subsequently treated with open reduction and internal fixation (ORIF) using a plating technique (shown in Fig. 1). Postoperatively, the patient and her family were explicitly instructed to avoid weight-bearing on the affected limb to ensure proper healing and prevent complications, but the insufficient fixation was due to the failure to recognize the fracture extension during the procedure.

**Fig. 2:**
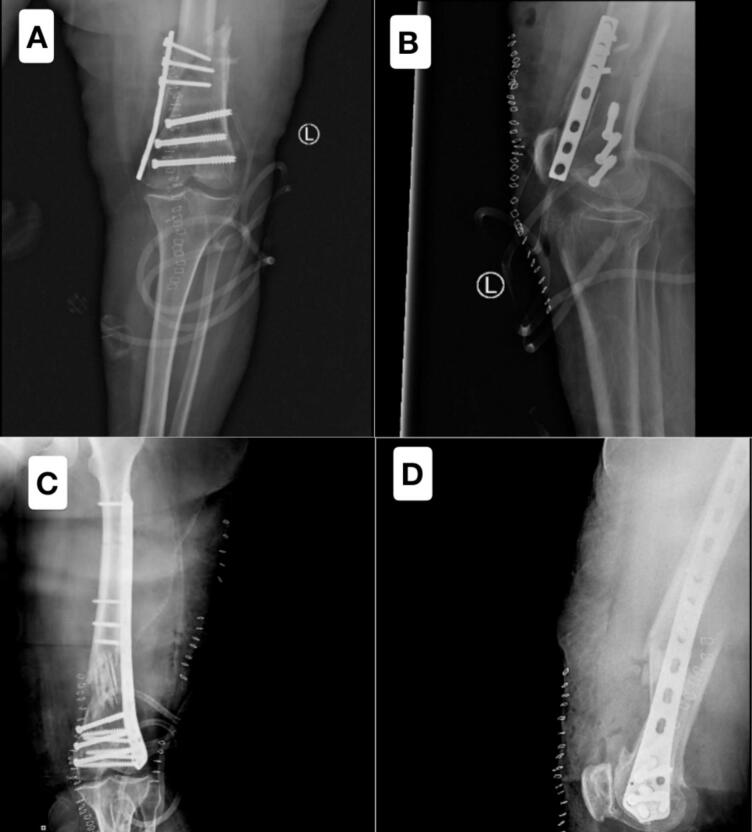
Figs. A and B illustrate the failure of the fixation caused by the patient's inadvertent weight-bearing on the operated limb. Figs. C and D display the postoperative X-rays following the revision surgery, during which the medial antigliding plate was removed and replaced with a long lateral distal femur locking plate, ensuring enhanced stability and improved alignment of the fracture site.

Two days postoperatively, while still admitted to the hospital, the patient, due to her cognitive impairment and associated mental health issues, failed to adhere to the medical team's instructions regarding non-weight-bearing on the operated limb. Consequently, she placed full weight on the affected leg, leading to a fall that resulted in failure of the fixation (shown in Fig. 2).

As a result, the patient underwent a revision surgery, during which the medial antigliding plate was removed and replaced with a long lateral distal femur locking plate to provide enhanced stability, the inadequate reduction resulted from the use of a bridging plate with functional reduction to preserve length, alignment, and rotation, rather than achieving anatomical reduction in the shaft fracture. Following the second surgery, the patient was kept under close observation in the orthopedic ward. Her postoperative care included regular dressing changes, physiotherapy exercises, and careful monitoring to ensure proper recovery.

A surgical drain was placed during the revision surgery and was removed on postoperative day 3, while surgical clips were removed on postoperative day 14. Serial follow-up X-rays were obtained to monitor fracture healing and fixation stability (shown in Fig. 2). The patient and her family were provided with clear instructions to avoid weight-bearing on the operated limb to prevent further complications.

Six weeks postoperatively, the patient presented with severe left thigh pain. After stabilizing her condition and administering appropriate analgesia, a thorough examination was performed. The evaluation revealed severe tenderness over the operated limb and complete inability to perform active movements, while the neurovascular examination remained intact.

**Fig. 3:**
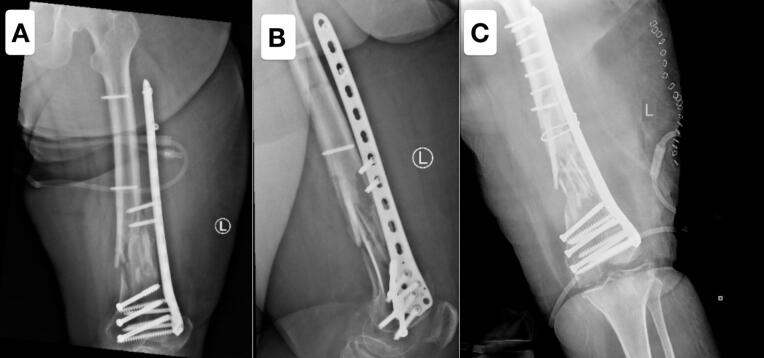
Figs. A and B demonstrate the failure of the fixation, characterized by proximal screw breakage through the plate, which compromised the stability of the construct. Fig. C illustrates the outcome following the revision surgery, during which the previous plate was removed and replaced with a new locking compression plate (LCP) secured with locking screws and cancellous screws. A cerclage wire was additionally utilized to enhance fracture reduction and stabilization.

Radiographic imaging (shown in Fig. 3) demonstrated failure of the fixation, with proximal screw breakage through the plate. In light of these findings, the patient underwent a revision surgery, during which the previous plate was removed and replaced with a new locking compression plate (LCP) secured with locking screws and cancellous screws. Additionally, a cerclage wire was utilized to aid in achieving proper fracture reduction and stabilization (shown in Fig. 3).

The patient and her family were provided with clear instructions to avoid weight-bearing on the operated limb to prevent further complications. One week after the revision procedure, the patient was discharged home with a regimen of oral antibiotics due to surgical site infection, instructions for regular wound dressing, and an appointment for follow-up in the outpatient orthopedic clinic.

## 3 Discussion

We present a case involving a 59-year-old female patient with an intellectual disability and a history of recurrent femoral fractures resulting from frequent falls. A femur fracture refers to a break in the femoral bone, which may also be classified as a hip fracture depending on the anatomical location. These fractures are particularly prevalent in elderly individuals, as age-related factors such as osteoporosis significantly weaken bone integrity. Among older adults, femoral fractures are associated with substantial morbidity, including increased mortality rates, diminished quality of life, and long-term physical impairments. Studies estimate that approximately 18 % to 28 % of older adults who sustain a femoral fracture succumb to complications within the first year following the injury [3].

With the global population aging, the incidence of femoral fractures is steadily rising, creating an urgent need for enhanced medical and nursing interventions tailored to the complexities of geriatric patients with multiple comorbidities. The management of femoral fractures in this population not only involves addressing the physical injury but also requires careful consideration of the patient's psychological and cognitive well-being. Psychiatric disorders are highly prevalent among older adults with femoral fractures, with delirium, depression, and dementia being the most common conditions observed. These psychiatric comorbidities can adversely affect surgical outcomes, prolong recovery, and complicate both the perioperative and postoperative phases of care. Additionally, cognitive impairments in this patient population often lead to challenges in communication and comprehension, which can hinder effective medical management and rehabilitation efforts [4].

The standard approach to managing femoral fractures includes surgical intervention, which may involve internal fixation techniques such as nailing, plating, or external fixation, followed by rigorous postoperative care and physical rehabilitation. While physical recovery has traditionally been the primary focus, emerging evidence highlights the significant role of psychological factors in influencing functional outcomes. For example, the risk of postoperative cognitive impairment, including delirium, can be mitigated through reorientation strategies, such as introducing familiar objects and maintaining a recognizable environment for the patient. Pain management is another critical component of care, as inadequate pain control can exacerbate psychological stress and delay mobilization. Furthermore, preventive measures aimed at reducing complications, such as infections, venous thromboembolism, and pressure ulcers, are essential to optimize recovery [[4], [5], [6]], also psychiatric and geriatric evaluation, social work involvement for caregiver support, and physiotherapy for rehabilitation. These measures are crucial in improving compliance, functional recovery, and overall patient outcomes.

The prognosis for elderly patients with femoral fractures is intricately linked to their psychological resilience, which has been identified as a key determinant of rehabilitation outcomes. Patients with lower levels of resilience are more likely to experience delayed recovery, prolonged hospital stays, and an elevated risk of postoperative complications, such as delirium and cognitive decline. Pre-existing mental impairments further compound these challenges, stressing the importance of comprehensive preoperative assessments and multidisciplinary care plans. Early mobilization and structured rehabilitation programs have demonstrated efficacy in improving functional outcomes and reducing the overall burden of disability in this patient population. Hence, a holistic approach that integrates physical, psychological, and social dimensions of care is paramount to achieving optimal outcomes [4,5,[7], [8], [9], [10]].

The success of distal femur fracture management depends not only on patient compliance but also on the adequacy of surgical fixation. In this case, insufficient initial fixation and inadequate fracture reduction contributed to recurrent failures. Biomechanical factors, such as implant selection, screw positioning, and load distribution, play a crucial role in achieving long-term stability. Studies suggest that long lateral locking plates, combined with adjunct stabilization techniques like cerclage wiring, improve outcomes in complex fractures. Additionally, a multidisciplinary approach, including psychiatric and geriatric evaluation, social support, and early physiotherapy, is essential in cognitively impaired patients to enhance adherence, prevent complications, and optimize recovery [[4], [5], [6]].

## 4 Conclusion

In conclusion, the management of distal femur fractures in patients with intellectual disabilities involves addressing both physical and cognitive factors to achieve better outcomes and reduce complications. This case shows the difficulties associated with non-compliance to postoperative care instructions and emphasizes the importance of tailored strategies, including enhanced patient education, close monitoring, and advanced fixation techniques, to prevent recurrent failures. Incorporating psychological and cognitive resilience into perioperative care plans plays a vital role in improving recovery and minimizing long-term disability in this vulnerable group.

## CRediT authorship contribution statement

• **Zaid Sawaftah**: Contributed to data collection, literature review, and drafting of the manuscript.
• **Ahmad R. Soboh**: Provided expertise in orthopedic aspects and contributed to the manuscript revision.
• **Ameer Awashra**: Coordinated the writing process, supervised the manuscript preparation, and reviewed the final draft for submission.
• **Osama Ewidat**: Assisted in conducting the literature search and contributed to manuscript drafting.
• **Alaa Hmeedan**: Conceptualization, Data curation, Writing - review & editing.
• **Qusai Jaludi**: Provided expertise in orthopedic aspects, Provided general input on the case report structure and critically reviewed the manuscript.

## Informed consent

Written informed consent was obtained from the patients for their anonymized information to be published in this article.

## Methods

The work has been reported in line with the SCARE criteria [7].

## Ethical approval

Our institution does not require ethical approval for reporting individual cases or case series.

## Guarantor

Ameer Awashra.

## Research registration number

1. .Name of the registry: Not applicable
2. Unique identifying number or registration ID: Not applicable
3. Hyperlink to your specific registration: Not applicable.

## Funding

No specific grant from funding agencies was received for this work.

## Declaration of competing interest

The authors state that they have no conflict of interest to be mentioned.
